# Competitive Endogenous RNA Network Involving miRNA and lncRNA in Non-Hodgkin Lymphoma: Current Advances and Clinical Perspectives

**DOI:** 10.3390/biomedicines9121934

**Published:** 2021-12-17

**Authors:** Mara Fernandes, Herlander Marques, Ana Luísa Teixeira, Rui Medeiros

**Affiliations:** 1 Molecular Oncology and Viral Pathology Group, Research Center of IPO Porto (CI-IPOP)/RISE@CI-IPOP (Health Research Network), Portuguese Oncology Institute of Porto (IPO Porto)/Porto Comprehensive Cancer Center (Porto.CCC), 4200-072 Porto, Portugal; mara.aires.fernandes@ipoporto.min-saude.pt (M.F.); ana.luisa.teixeira@ipoporto.min-saude.pt (A.L.T.); 2 Research Department of the Portuguese League against Cancer Regional Nucleus of the North (LPCC-NRN), 4200-177 Porto, Portugal; 3 Faculty of Medicine, University of Porto (FMUP), 4200-319 Porto, Portugal; 4 Life and Health Sciences Research Institute (ICVS), School of Medicine, Campus de Gualtar, University of Minho, 4710-057 Braga, Portugal; herlandermarques@hotmail.com; 5 ICVS/3B’s–PT Government Associate Laboratory, 4805-017 Braga/Guimarães, Portugal; 6 Department of Oncology, Hospital de Braga, 4710-243 Braga, Portugal; 7 CINTESIS, Center for Health Technology and Services Research, Faculty of Medicine, University of Porto, 4200-450 Porto, Portugal; 8 ICBAS—Instituto de Ciências Biomédicas Abel Salazar, Universidade do Porto, 4050-513 Porto, Portugal; 9 Biomedical Research Center (CEBIMED), Faculty of Health Sciences of Fernando Pessoa University (UFP), 4249-004 Porto, Portugal

**Keywords:** lymphoma, non-Hodgkin’s lymphoma, miRNAs, lncRNAs, biomarkers

## Abstract

Non-Hodgkin lymphoma (NHL) is a heterogeneous malignancy with variable patient outcomes. There is still a lack of understanding about the different players involved in lymphomagenesis, and the identification of new diagnostic and prognostic biomarkers is urgent. MicroRNAs and long non-coding RNAs emerged as master regulators of B-cell development, and their deregulation has been associated with the initiation and progression of lymphomagenesis. They can function by acting alone or, as recently proposed, by creating competing endogenous RNA (ceRNA) networks. Most studies have focused on individual miRNAs/lncRNAs function in lymphoma, and there is still limited data regarding their interactions in lymphoma progression. The study of miRNAs’ and lncRNAs’ deregulation in NHL, either alone or as ceRNAs networks, offers new insights into the molecular mechanisms underlying lymphoma pathogenesis and opens a window of opportunity to identify potential diagnostic and prognostic biomarkers. In this review, we summarized the current knowledge regarding the role of miRNAs and lncRNAs in B-cell lymphoma, including their interactions and regulatory networks. Finally, we summarized the studies investigating the potential of miRNAs and lncRNAs as clinical biomarkers, with a special focus on the circulating profiles, to be applied as a non-invasive, easy-to-obtain, and reproducible liquid biopsy for dynamic management of NHL patients.

## 1. Introduction

Non-Hodgkin lymphomas (NHL) are a very heterogeneous group of lymphoproliferative malignancies characterized by the infiltration of lymphoid tissues [1]. The majority of NHL are derived from B cells (85% to 90%), while the remaining are derived from T cells or NK cells [1]. The most common NHL subtypes are the aggressive diffuse large B-cell lymphoma (DLBCL) (~30%) and the indolent follicular lymphoma (FL) (~20%) [1]. Over the years, with the advance of gene-expression profiling and next-generation sequencing, new evidence has provided substantial insights into the etiology and the molecular alterations of the different entities comprising NHL; however, several questions remain unanswered; therefore, it is crucial to deepen our understanding of the pathogenesis of NHL in order to adopt a more personalized therapeutic approach for these patients.

In general, treatment modalities of NHL encompass chemotherapy, immunochemotherapy, and/or radiation therapy. In fact, the addition of rituximab to the anthracycline-containing chemotherapy regimen (cyclophosphamide, doxorubicin, vincristine, prednisone–R-CHOP) was a major turning point in NHL patients’ management, significantly improving the outcomes. However, a significant percentage of patients (approximately 20–50%) are refractory ab initio or ultimately relapse, presenting only a 20–40% 2-year overall survival rate [2,3,4]. These raise the urgent need to broaden our knowledge concerning new molecular and biological biomarkers with predictive and prognostic potential, and ultimately, develop new therapeutic agents.

Recently, non-coding RNAs (ncRNAs), which were once thought to be “junk RNA”, have emerged as essential players in the molecular events of normal B-cell development and in lymphomagenesis [5]. MicroRNAs (miRNAs) are undoubtedly the class of ncRNAs most studied over the years, especially due to their relevant biological function in gene regulation [6]. MiRNAs are characterized as small ncRNAs with ~22 nucleotides in length, present in all eukaryotic cells, and highly conserved. They function as gene regulators at a post-transcriptional level through binding to the 3′ untranslated region (UTR) of a target mRNA, which results in their repression or degradation [7]. Recently, the role of miRNAs as regulatory players in B-cell lymphomas is being unveiled, and they have been proposed as potential biomarkers for the diagnosis, prognosis, and prediction of therapy response [8]. To date, it is established that miRNAs can be found in circulation, not only in its cell-free form but also encapsulated in extracellular vesicles (such as exosomes), which permit them to function in a paracrine manner during lymphoma development and progression (reviewed by Fernandes et al. [9]).

**Figure 1:**
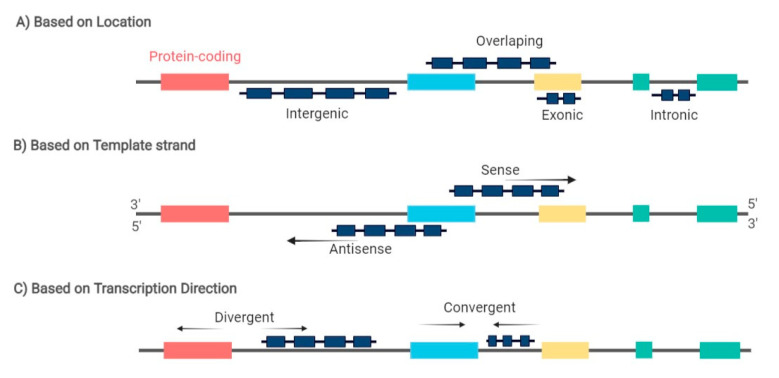
LncRNA can be classified based on: (**A**) the genomic location between two coding genes in: intronic, exonic, intergenic, and overlapping lncRNA; (**B**) the template strand from which they are transcribed in: sense and antisense lncRNA; and (**C**) the direction of lncRNA transcription in: divergent and convergentlncRNA. Arrows indicate the transcription direction. Red, blue, yellow, and green boxes represent exons from different coding genes.

Recent studies have shown that another class of ncRNAs, known as long non-coding RNAs (lncRNAs), are also master regulators of multiple protein-coding genes and are involved in all cancer hallmarks [10,11]. LncRNAs are characterized for being more than 200 nucleotides long and can be further classified based on their biogenesis loci in intronic, exonic, intergenic, or overlapping sense/antisense lncRNAs, and divergent/convergent lncRNAs (Figure 1) [12]. These molecules exhibit relatively low expression but high tissue and disease-specific expression patterns [13]. Among the different functions of lncRNAs in gene expression regulation is the remarkable interplay between lncRNAs and miRNAs, which has the ability to balance miRNA function as miRNA sponges/decoys, creating a competitive endogenous RNA (ceRNA) network [14,15]. LncRNAs can sequester miRNAs by presenting biding sequences for miRNAs and impairing their functional interaction with mRNA [16]. Moreover, one lncRNA has the ability to sponge various miRNAs through different biding sites, as seen, for example, for lncRNA MALAT1, which was demonstrated to target miR-101, miR-129, and miR-199a [17,18,19,20]. Therefore, the miRNA regulatory network is more intricate than previously thought by adding another regulatory layer to the network involving lncRNAs. Recent studies have shown that lncRNAs regulate cell differentiation, and their deregulation plays a key role in the pathogenesis of cancer [21,22]. In fact, some studies have been analyzing the expression pattern of lncRNAs in the different B-cell lymphoma subtypes. However, compared to solid tumors, there is still a limited number of studies analyzing the role of lncRNAs during normal B-cell development and as key players in B-cell malignancies.

In this review, we will address the current knowledge of the biological function of miRNAs and lncRNAs in B-cell development and how this intricate regulatory network involving the two ncRNAs plays a central role in lymphomagenesis. Moreover, we attempt to shed light on the potential of miRNAs and lncRNAs as biomarkers to be used in the liquid biopsy-based clinical management of NHL patients in order to potentiate treatment efficacy.

## 2. MiRNAs and lncRNAs Deregulation in Lymphomagenesis

### 2.1. Evidence Acquisition

The literature search was performed in the PubMed database and Google Academic to identify articles published between January 2000 and July 2021 that analyzed the role of miRNAs and lncRNAs in B-cell lymphoma using the following search terms: ((“Non-coding RNA”) OR (“microRNA” OR “miRNA” OR “miR”) OR (“lncRNA” OR “long noncoding RNA”) AND (“lymphoma” OR “B-cell Lymphoma” OR “NHL”). In order to search for studies investigating miRNAs and lncRNAs as a diagnosis, subtype, treatment response, or prognosis biomarkers, with special focus in circulating biomarkers, the following search terms were used: (“B-cell Lymphoma” OR “Lymphoma Non-Hodgkin” OR “NHL”) AND ((miRNA OR microRNA OR miR) OR (“long non-coding RNA” OR lncRNA)) AND (Biomarker OR Biomarkers) AND (circulating OR “peripheral blood” OR “cell-free” OR free OR plasma OR serum). We excluded articles not published in English, reviews, case reports, opinion articles, as well as those studies carried out on other diseases.

### 2.2. The Role of miRNAs in B-Cell Lymphomagenesis

**Figure 2:**
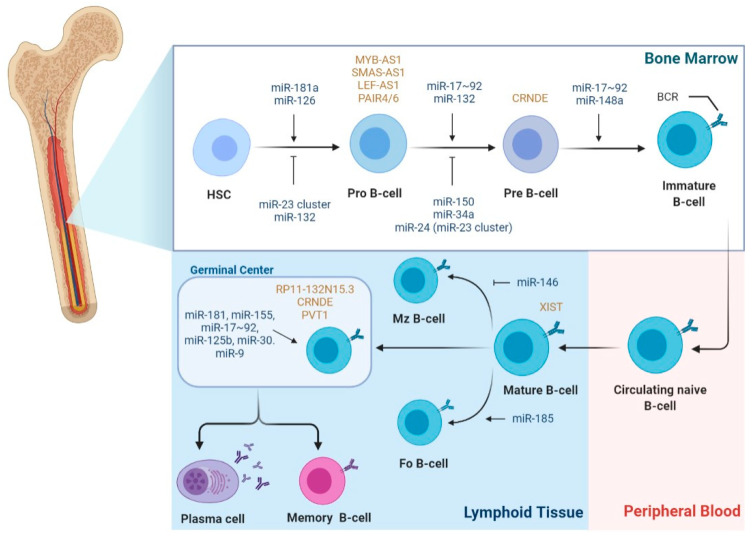
miRNA and lncRNA expression during the different stages of B-cell development. During B-cell development, miRNAs and lncRNAs show a stage-specific expression pattern. For example, miR-181a-5p, miR-150-5p, miR-132-3p, and miR-126-3p were shown to be differentially expressed during the development stages of B cells; in particular, miR-181a-5p ectopic overexpression in common lymphoid progenitors results in an increasing total number of B cells. Conversely, overexpression of miR-23a-5p in HSCs results in the inhibition of B-cell development. MiRNAs are involved in the modulation of the checkpoint of pro to pre-B-cell transition. MiR-132-3p shows a stage-specific and BCR-dependent expression, being normally expressed after the pro-B stage; miR-24-3p, miR-34a, and miR-150-5p, when overexpressed, block the transition at pro to pre-B-cell. In secondary lymphoid tissues, miR-155 and miR-181b are highly expressed in activated B-cells in germinal centers. miR-155 and miR-181b-deficient B cells have defective antibody class switching and differentiation into plasma cells; both miRNAs target activation-induced cytidine deaminase (AID) and PU.1, which promote antibody class switching and antibody production. Other miRNAs, e.g., miR-9, miR-125b, and the miR-30 family, are expressed in GC B cells and enhance plasma-cell differentiation. Concerning lncRNAs regulation of B-cell development, lncRNAs MYB-AS1, SMAS-AS1, and LEF-AS1 were found to play a role in early B cells; CRNDE is overexpressed during proliferating stages, such as pre-B-cells and centroblasts in the GC. LncRNA XIST modulates the X-linked gene regulation from antigen naïve B-cells to activated B-cells during B-cell stimulation. Expression of lncRNAs PVT1 and RP11-132N15.3 were associated with the expression of AID in the GC. (Abbreviations: B-cell receptor (BCR); Follicular B cells (FO B-cells); Hematopoietic stem cells (HSCs); Marginal zone B-cells (MZ B-cells)).

Considering that B-cell development is a highly regulated process, it is not surprising that miRNAs have been implicated in the regulation of most of the stages comprising this process (Figure 2). Interestingly, during B-cell development, most miRNAs show a stage-specific expression pattern, highlighting their stage-specific function [23]. The process involving B-cell differentiation seems to be prone to malignant transformation, with increasing evidence showing that disruption of the miRNA network takes part in the initiation and maintenance of lymphomagenesis.

Given the regulatory role of the miR-17~92 cluster (comprising miR-17, miR-18a, miR-19a, miR-19b-1, miR-20a, and miR-92-1) during the stages of bone marrow (BM) B-cell development and central tolerance, its deregulated expression was shown to have oncogenic potential [24]. In fact, the 13q31.3 locus, which encodes this cluster, is often found amplified in B-cell malignancies, including DLBCL, mantle cell lymphoma (MCL), and FL [25,26,27]. The overexpression of the miR-17~92 cluster, concomitantly with the expression of oncogene Myc, promoted the onset and increased tumor growth in a mouse model of B-cell lymphoma [28]. Interestingly, Xiao et al. generated mice expressing two copies of human miR-17~92 in the B and T-cell compartments and observed that these mice developed lymphoproliferative disease and autoimmunity, showing elevated autoantibody and lymphocyte infiltration into non-lymphoid organs. The effects of miR-17~92 upregulation seemed to be associated with decreased levels of BIM and PTEN (both miR-17~92 targets), resulting in increased proliferation and resistance to cell death [29]. Moreover, a study identified miR-19a and miR-19b as the key oncogenic components of the miR-17~92 cluster. MiR-19 was shown to target PTEN, resulting in the activation of the AKT1-MTOR pathway and ultimately promoting cell survival in vitro and boosting tumor formation in vivo [30]. A B-cell-specific miR-17~92 transgenic mouse developed lymphomas with high penetrance, phenotypically resembling human lymphomas, including DLBCL, showing the role of the miR-17~92 cluster as a driver of B-cell lymphomagenesis [31]. The cell-cycle regulators E2F3 and BIM were identified as direct targets of the miR-17~92 cluster; therefore, they are involved in the mediation of B-cells’ proliferation and survival. Additionally, miR-17~92-driven lymphoma cells exhibited constitutive activation of PI3K due to PTEN and PHLPP2 repression, and of the NF-κB pathway, due to inhibition of NF-kB deubiquitinases, CYLD, and A20 [31]. Lastly, chemical inhibition of PI3K (AZD8055) or NF-kB (BMS-345541) resulted in reduced tumor size and prolonged survival of lymphoma-bearing mice, highlighting the importance of the miR-17~92 cluster as a driver of both tumor development and maintenance [31].

Another central miRNA during B-cell development, which is also aberrantly expressed in different B-cell lymphomas, is miR-155. MiR-155-5p controls plasma-cell production by inhibiting transcription factor SPI1, culminating in the downregulation of PAX5, which is required for normal terminal B-cell differentiation [32]. In vitro and in vivo inhibition of miR-155 was shown to diminish cell survival and cell proliferation and reduce tumor growth in mice [33]. Thai et al. observed a reduction in the total number of germinal center (GC) B cells in miR-155-deficient mice and, conversely, an increase in knock-in mice, probably as a result of targeting the transcription factor SPI1 [34,35]. MiR-155 was reported to be involved in the modulation of the PIK3CA-AKT1 pathway by directly targeting the negative regulator PIK3R1 (p85α) in DLBCL cells [36]. According to Zhu et al., miR-155 seems to modulate cell proliferation, cell cycle, and apoptosis of DLBCL cells by targeting transforming growth factor-β receptor 2 (TGFBR2) [37]. Several studies have been focusing on unveiling the miR-155 oncogenic role in B-cell lymphomas, reporting that its targets NIAM, histone deacetylase 4 (HDAC4), and SHIP1 [38,39,40]. Interestingly, Rai et al. reported a unique mechanism in DLBCL cells involving miR-155 overexpression, which renders lymphoma cells the ability to escape TGF-β’s growth-inhibitory effects. They reported that miR-155 directly targets the bone morphogenetic protein (BMP)-responsive transcriptional factor SMAD5 and that TGF-β1 also activated SMAD5 in DLBCL cells. Thus, the overexpression of miR-155 resulted in resistance to the cytostatic effects derived from both BMPs and TGF-β1 due to impaired activation of p21 and impaired cell cycle arrest [41]. During follicular B-cell maturation, miRNAs firmly regulate class-switch recombination, which includes further Ig gene rearrangements. miR-125b-5p targets transcription factors PRDM1 and IRF4, both involved in the activation of class-switch recombination, making its repression required for the normal B-cell development; otherwise, the development of B-cell malignancies may occur [42,43,44]. Moreover, similar results were obtained by other miRNAs, namely miR-30b-5p, miR-30d-5p, and miR-9-5p, by also targeting PRDM1 [45].

In GC, miR-21 promotes B-cell activation and proliferation, driving GC B-cell expansion and response [46]. The role of miR-21 as oncomiRNA in lymphoma was demonstrated using a mouse model overexpressing miR-21, which resulted in the spontaneous development of a pre-B-cell lymphoma. Inversely, tumor regression was observed after miR-21 repression due to increased apoptosis and proliferation arrest [47]. Moreover, PTEN and PDCD4 have been indicated as miR-21 targets and regulators of the PI3K/AKT and MAPK signaling pathways, which in turn mediate proliferation and apoptosis in lymphoma cells [48,49]. Conversely, miR-21 expression was shown to be transcriptionally activated by NF-κB [50]. Another described oncomiRNA in B-cell lymphomas is miR-217, whose expression is found upregulated as a result of B-cell stimulation in the context of the GC reaction. GC reaction is induced by miR-217 due to stabilization of Bcl-6 expression, resulting in enhanced production of class-switched antibodies and frequency of somatic hypermutation [51]. Importantly, Yébenes et al. showed that miR-217 overexpression also promotes mature B-cell lymphomagenesis. These results suggest that both miR-217 and Bcl-6 participate in the same network that makes GC cells permissive to genomic instability and prone to malignant transformation [51].

On the other hand, the deregulation of tumor-suppressive miRNAs is also frequently implicated in both B-cell lymphoma development and progression. In this instance, miR-34a has emerged as a tumor suppressor in lymphoma, linked to the p53 network. After p53 stabilization, miR-34a is upregulated in B cells, leading to a direct decrease of B-MYB and indirect decrease of E2F1 and the induction of cell-cycle arrest [52]. Moreover, p53 FOXP1 has also been described as a miR-34a target, where miR-34a downregulation results in higher levels of FOXP1 and BCL6, which in turn supports DLBCL proliferation [53,54,55]. Furthermore, miR-34a was also shown to target AXL, a receptor tyrosine kinase that activates PI3K/AKT signaling, and to be overexpressed in chronic lymphocytic leukemia (CLL) [56]. Cluster 15/16 miRNAs’ implication in the pathogenesis of B-cell lymphomas was first demonstrated when Calin et al. reported that miR-15/16 downregulation is due to the loss of the 13q14 locus, where the cluster is encoded in CLL [5]. Subsequent studies demonstrated that their tumor suppressor function was, at least in part, through the activation of cell apoptosis by targeting BCL2, MCL1, and CDK6 [57]. Moreover, miR-15/16 were shown to be part of a positive feedback loop involving TP53, which is a direct target of the cluster in CLL [58]. The deregulation of the miR-15/16 cluster has also been linked with the pathogenesis of other types of lymphoma, such as MCL, where their downregulation was induced by c-Myc and histone deacetylase [59].

Another MYC-regulated miRNA that has been implicated in B-cell pathogenesis is miR-150. Presenting a stage-specific expression during B-cell development, miR-150-5p prevents pro to pre-B-cell transition when overexpressed prematurely [60,61]. By downregulating miR-150, MYC induces the expression of FOXP1 and GAB1, regulators of cell survival and B-cell receptors, and of NF-κB signaling in malignant B cells [62]. The deregulation of miRNAs has also been implicated in other signaling pathways of B-cell lymphomas, such as mitogen-activated protein kinase (MAPK) and Wnt/β-catenin signaling pathways. For example, miR-101 was shown to regulate cell proliferation and apoptosis by targeting mitogen-activated protein kinase 1 (MEK1), an upstream protein kinase of the ERK/MAPK pathway, in DLBCL [63].

Taken together, these studies have been confirming the causative role of deregulated miRNA expression in B-cell lymphomagenesis, identifying potential clinically relevant miRNAs for profiling studies and relevant targets for miRNA-based therapies.

### 2.3. The Role of LncRNAs in B-Cell Lymphomagenesis

Regarding the regulatory role of lncRNA during the different stages of B-cell development and as drivers of B-cell malignancies, there is still scarcer information when compared to miRNAs. LncRNA expression profiling studies have reported that lncRNA exhibits cell-type-specific expression patterns during the different stages of B-cell differentiation (Figure 2) [64,65,66,67]. Consequently, each B-cell subset can be differentiated using its unique lncRNA expression profile [67]. Petri et al., using a guilt-by-association method, analyzed lncRNAs originated from protein-coding genes with known functions in B-cell development and identified antisense lncRNAs, such as MYB-AS1, SMAS-AS1, and LEF-AS1, with roles in early B cells, associated with RAG2, VPREB1, DNTT, LEF1, SMAD1, and MYB expression [66]. On the other hand, lncRNA colorectal neoplasia differentially expressed (CRNDE) showed high expression during the proliferating stages, such as pre-B cells and centroblasts in the GC, which was tightly associated with the expression of mitotic cell cycle genes [66]. In fact, CRNDE was previously demonstrated to be linked to cell-cycle and metabolic changes during proliferation [68,69]. Brazão et al. identified the expression of PVT1 and some uncharacterized lincRNAs, such as LINC00487, LINC00877, and RP11-132N15.3, associated with the expression of AID and SERPINA9, both specifically expressed in GC centroblasts and centrocytes. Of note, RP11-132N15.3 is described to be encoded approximately 240 kilobases upstream of BCL6 [65,66]. Additionally, based on mice models, several lncRNAs demonstrated a PAX5-dependent expression, a transcription factor involved in B-cell commitment, which were shown to be bound by PAX5 and to have human orthologs previously described [65]. Verma-Gaur et al. proposed that germ-line-transcribed lncRNAs regulate V(D)J recombination during the pro-B stage by assisting locus compaction, which brings heavy chain genes close to undergoing efficient gene rearrangements. Remarkably, the most expressed transcripts were PAX5-activated intergenic repeat (PAIR) elements, PAIR4 and PAIR6, whose transcription is antisense to PAX5 [70]. Moreover, they demonstrated that YY1^−/−^ pro-B cells, a key player in distal VH gene rearrangement, presented a considerable reduction in antisense transcription between the PAIR promoters and the intronic enhancer, and consequently an impaired distal VH gene rearrangement, reinforcing the hypothesis of PAIR4 and PAIR6 as central regulators of the V(D)J recombination [70]. Later on, Syrett et al. proposed that YY1 binds and relocates lncRNA Xist to the inactive X-chromosome during B-cell stimulation, modulating the X-linked gene regulation from antigen naïve B cells to activated B cells [71]. According to Tayari et al., naïve and memory B-cell subsets exhibit analogous lncRNA expression patterns but are distinct from highly proliferating GC-B cells, which was corroborated in two other profiling studies [64,65,67].

Non-coding antisense transcripts of PU.1 were reported to inhibit the expression of PU.1 at a translation level, which could indicate its pivotal role in lymphomagenesis given the regulatory function of PU.1 in B-cell differentiation [72,73].

On the other hand, over the past few years, some studies have been trying to unveil the mechanistic pathways associated with the deregulation of lncRNAs during lymphomagenesis. In this instance, TP53 has been linked to the expression of some lncRNAs in different lymphoma subtypes. In fact, Blume et al. demonstrated, for the first time, the association of lncRNAs and the p53 pathway in CLL and lymphoma by inducing a p53-dependent DNA damage response, which resulted in increased expression of two lncRNAs, NEAT1 and lincRNA-p21, regulating apoptosis or cell-cycle arrest and DNA repair [74]. In DLBCL, p53 can directly bind to the promoter region of the lncRNA PANDA, which inactivates the MAPK/ERK signaling pathway, suppressing the proliferation of DLBCL cells by a G0/G1 cell-cycle arrest [75]. Peng et al. demonstrated that lncRNA HULC regulates DLBCL cell apoptosis and cell proliferation via the upregulation of antiapoptotic BCL2 protein and cyclin D1 [76]. The Wnt/β-catenin signaling pathway was shown to be activated by lncRNA FIRRE through promoting the nuclear translocation of β-catenin [77]. LncRNA DBH-AS1, found to be upregulated in DLBCL, was identified as a positive regulator of cell proliferation, migration, and invasion via binding to the RNA-binding protein BUD13 homolog (BUD13), which in turn regulates fibronectin 1 expression [78]. Cheng et al. observed suppression of DLBCL cell proliferation and tumor growth after TUG1 knockdown, promoting the ubiquitination of MET and the subsequent degradation [79]. Similarly, the knockdown of HOTAIR expression resulted in growth inhibition, cell-cycle arrest, and apoptosis, possibly involving the regulation of PI3K/AKT/NF-κB pathways [80]. Moreover, the HOTAIR function was associated with epigenetic regulation by recruiting polycomb repressive complex 2 (PRC2) proteins (EZH2, SUZ12, and EED), which induces H3K27me3, strongly related with aggressive DLBCL [81]. Sehgal et al. described an alternative splicing mechanism of Fas, firmly regulated by its antisense lncRNA FAS-AS via binding to RBM5, which induces Fas-mediated apoptosis in lymphoma. Conversely, FAS-AS1 expression is regulated by EZH2, which hyper-methylates the FAS-AS1 promoter, suppressing its expression [82]. In MCL, EZH2 has also been associated with the lncRNA MALAT1. Specifically, Wang et al. demonstrated that the knockdown of MALAT1 resulted in reduced levels of EZH2 and H3K27me3, while the expression levels of CDKs inhibitors p21 and p27 increased, resulting in cell-cycle arrest at the G1/S transition [83]. Similarly, a study by Li et al. reported silencing MALAT1 in DLBCL cells results in decreased cell proliferation and invasion and increased cell apoptosis. Concomitantly, they observed an increase in the expression levels of two autophagy indicators, LC3-II/LC3-I proteins and p62, reflecting the increase in autophagy in DLBCL cells [84]. A previously identified NOTCH1-regulated lncRNA in T-ALL, LUNAR1, was shown to regulate cell cycles and proliferation in lymphoma cells. LUNAR1 downregulation resulted in the inhibition of cell proliferation via E2F1, cyclin D1, and p21 [85,86].

Recently, emerging evidence has been demonstrating that a large group of lncRNAs exert their function as ceRNAs to fine-tune the miRNA-mediated regulation of protein abundance in lymphoma development.

### 2.4. The Role of LncRNAs as ceRNAs in B-Cell Lymphomagenesis

Karreth et al. demonstrated that lncRNA BRAFP1, which is aberrantly expressed in B-cell lymphomas, acts as a ceRNA with BRAF mRNA, increasing its stability and BRAF levels by sequestering specific BRAF-targeting miRNAs, such as miR-134, miR-543, and miR-653. Consequently, BRAF activates MAPK signaling, resulting in DLBCL cells’ proliferation [87]. In fact, NEAT1 was identified as an MYC-regulated transcript promoting DLBCL cells proliferation and lymphomagenesis by regulating the miR-34b-5p-GLI1 pathway [88]. Interestingly, NEAT1, along with LincRNA-p21, were also identified as p53-dependent DNA damage response machinery in lymphoma and CLL [74]. Zhao et al. reported that lncRNA PEG10 exhibits an oncogenic role in DLBCL progression by sponging miR-101-3p, which directly targets KIF2A. Moreover, abrogation of PEG10 or KIF2A expression resulted in the inhibition of cell proliferation, migration, and invasion, and promotion of cell apoptosis [89]. Recently, Tian et al. reported the upregulation of PCAT1, a proven key carcinogenic driver of hepatocellular carcinoma, in DLBCL [90]. Moreover, they investigated the mechanism behind PCAT1 action in DLBCL cells and observed an interplay between this lncRNA and miR-508-3p, which the inhibition of results in upregulation of NFIB, inducing DLBCL cell proliferation, migration, and invasion [90]. Similarly, lncRNA UCA1 was found upregulated in DLBCL, being involved in the regulation of cell proliferation, migration, and invasion by competitively binding with miR-331-3p [91].

Another reported upregulated lncRNA in DLBCL is MALAT1, whose ceRNA function is through sponging miR-195, resulting in the activation of the immune checkpoint molecule PD-L1 and consequently promoting cell proliferation, migration, and immune escape. Moreover, MALAT1 can induce CD8+ T cell apoptosis and epithelial–mesenchymal transition (EMT)-like processes by regulating the Ras/ERK signaling pathway [92]. In MCL, the knockdown of MALAT1 resulted in cell-cycle arrest and impaired proliferation due to the upregulation of p21 and p27 by EZH2 [83]. MiR-423-5p was reported to be involved in a ceRNA network with lncRNA FOXP4-AS1 in MCL cells. Mechanistically, FOXP4-AS1 acts as a sponge to miR-423-5p, upregulating the expression of NACC1, which results in MCL cell proliferation, migration, and invasion [93].

The induction of proliferation and cell-cycle progression accompanied with inhibition of cell apoptosis was observed as a result of lncRNA LINC01857 upregulation through targeting miR-141-3p/MAP4K4 axis in DLBCL. The biological effect of LINC01857 was associated with the activation of the PI3K/mTOR pathway and the induction of the EMT process [94]. Huang et al. reported that lncRNA LINC00857 regulates the miR-370-3p/CBX3 axis by competing for miR-370-3p binding, resulting in the modulation of DLBCL cell proliferation and apoptosis [95]. The silencing of lncRNA SBF2-AS1 resulted in decreased cell viability and growth via directly targeting miR-494-3p and, consequently, resulting in decreased FGFR2 expression [96]. Recently, Xu et al. reported that transcription factor PAX5 activates lncRNA ARRDC1-AS1, which functions as a sponge for miR-2355-5p to upregulate ATG5, resulting in increased autophagy and progression of DLBCL [97]. Li et al. demonstrated the oncogenic function of TUC338 in DLBCL by sponging miR-28-5p, which leads to the activation of the EGFR/PI3K/AKT pathway [98]. Another recently identified oncogenic lncRNA in DLBCL is LINC00908, which, when silenced, results in the inhibition of cell proliferation and invasion. Moreover, LINC00908 function seems to be through directly binding and inhibiting miR-671-5p [99].

On the other hand, SMAD5-AS1 was reported to be downregulated in DLBCL since it inhibits DLBCL proliferation by sponging miR-135b-5p to ultimately upregulate APC expression and inhibit the Wnt/β-catenin pathway [100]. LINC00963 was also described as having a tumor-suppressive role in DLBCL cells since overexpression of this lncRNA resulted in the inhibition of cell proliferation in vitro and tumor growth in vivo. LINC00963 directly binds to miR-320a, regulating endoplasmic reticulum stress and autophagy via XBP-1 targeting [101].

An emerging class of lncRNA, known as small nucleolar RNA host genes (SNHGs), has been proposed to also function as miRNA sponges in B cell lymphoma. Chen et al. observed that SNHG12 knockdown inhibited cell growth, migration, and invasion in vitro and in vivo by sequestering miR-195 [102]. SNHG14 was reported to promote DLBCL progression and immune evasion by a positive feedback loop involving the SNHG14/miR-5590-3p/ZEB1 axis. Specifically, SNHG14 sequesters miR-5590-3p, upregulating ZEB1, which, in turn, transcriptionally activates SNHG14 and PD-L1, thus modulating the PD-1/PD-L1 checkpoint [103]. Similarly, another study also reported the role of SNHG14 in promoting both DLBCL progression and immune evasion via the PD-1/PD-L1 checkpoint, but in this instance by sponging miR-152-3p [104]. Zhu et al. demonstrated that mechanistically SNHG16 acts as a ceRNA by sequestering miR-497-5p, which targets PIM1 resulting in the promotion of proliferation and inhibition of apoptosis in DLBCL [105]. Moreover, lncRNA SNHG8 was described as having a cancer-promoting effect on DLBCL by directly targeting miR-335-5p, which promotes proliferation while inhibiting the apoptosis of DLBCL cells [106].

The recent emerging data concerning the action of lncRNAs as miRNAs sponges highlights the complex post-transcriptional balance involved in gene-regulation networks. Altogether, these studies show the significant role of ceRNAs in lymphoma pathogenesis, which needs further characterization.A better understanding of ceRNA interactions may open the opportunity to exploit this crosstalk for biomarkers discovery and precise RNA-based therapies in lymphoma.

## 3. MiRNAs and lncRNAs as Potential Biomarkers for NHL

**Figure 3:**
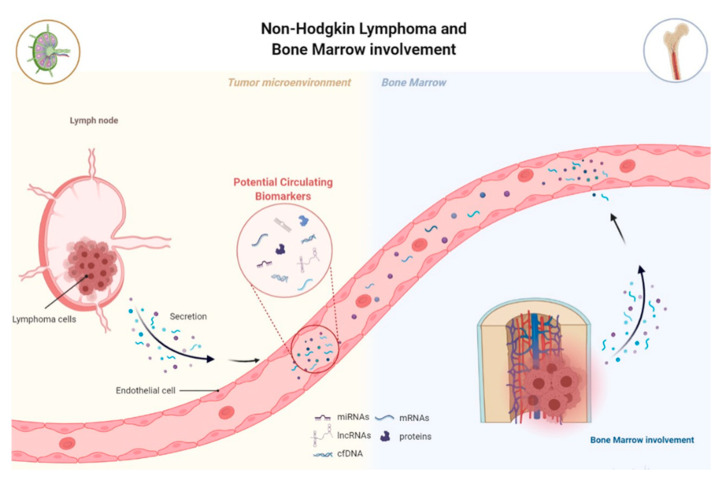
Lymphoma-related circulating-free DNA, RNA, or proteins are released by lymphoma cells into circulation, known as tumor circuloma. Analysis of the tumor circuloma can provide a non-invasive approach to screen, diagnose, and surveillance patients during the course of the disease. Moreover, the use of liquid biopsy in substitution of BM biopsy to detect BM infiltration by lymphoma opens the need to readdress the value of the routine standard analysis.

The current gold standard for NHL diagnosis and classification remains tissue biopsies of an involved lymph node (or a tumor in another organ) and BM aspiration and biopsy to establish BM involvement [1]. However, these procedures are not only invasive but also do not reflect tumor heterogeneity and clinical behavior. BM biopsy raises the question of its routine use, especially in patients with a low probability of bone marrow involvement, but also, being the collection sites only the sternum or ilium; they do not necessarily reflect the overall condition of the BM. Lastly, biopsies are unsuitable for dynamic monitoring during treatment and follow-up. The presence of circulating tumor-associated components, known as “tumor circulome”, which can be easily assessed, appears as a potential option as cancer biomarkers for liquid biopsies (Figure 3) [107]. One of the major components of “tumor circulome”, highly present in circulation, are the miRNAs [108]. MiRNAs emerged as excellent biomarker candidates due to their high stability in biological samples and their high specificity and sensitivity (Table 1) [8]. Similarly, increasing evidence has proposed lncRNAs are promising cancer diagnostic and prognostic biomarkers, especially given their high cell type, tissue, and disease type-specific expression (Table 2). Moreover, lncRNAs have been considered stable and can also be detected in circulation [109]. However, the majority of studies analyzing deregulated lncRNAs in lymphoma have been performed on tissue samples and cell lines.

### 3.1. MiRNAs and lncRNAs as Non-Invasive Diagnostic Biomarkers

A current clinical problem in the context of NHL remains the late and imprecise diagnosis, which in turn negatively conditions patient morbidity and mortality. Several authors have proposed and showed the potential of both individual miRNAs and lncRNAs or panels of these ncRNAs, as biomarkers for diagnosis and subclassification of NHL. Regarding the diagnostic potential, studies have analyzed the expression of miRNAs by comparing NHL cases to healthy controls in order to identify a specific miRNA signature. Lawrie et al.’s study showed, for the first time, that tumor-associated miRNAs, miR-155, miR-210, and miR-21 expression levels were upregulated in DLBCL patients’ serum compared to healthy controls [110]. Beheshti et al. first established a miRNA expression profile associated with DLBCL development based on cell lines and patient-derived xenograft models, which was then validated in patients’ serum. In the validation study, their profile was reduced to five miRNAs, let-7b, let-7c, miR-18a, miR-24, and miR-15a, which had the ability to discriminate DLBCL patients from healthy donors, with an accuracy of 91% [116,142]. Another study showed a 4-miRNA panel, comprising miR-15a, miR-16-1, miR-29c, and miR-155, significantly elevated in DLBCL serum and one downregulated, miR-34a, when compared to healthy controls [111]. More recently, Yuan et al. demonstrated a correlation between circulating levels of eight miRNAs and their matched formalin-fixed, paraffin-embedded (FFPE) samples [124]. Inada et al. demonstrated that the expression levels of circulating miR-15a-3p, miR-21-5p, miR-181a-5p, and miR-210-5p were significantly different between DLBCL patients and healthy controls, and specially miR-15a-3p, and miR-21-5p showed higher power to discriminate patients from controls [115]. MiR-34a-5p, miR31-5p, miR-155-5p, miR-150-5p, miR-15a-3p, and miR-29a-3p were shown to be elevated in CLL patients compared with healthy individuals and seemed to be associated with B-cell differentiation and homeostasis [127]. Most recently, Jørgensen et al. addressed the question of if it is possible to detect specific circulating miRNAs years before the diagnosis of B-cell lymphoma. Firstly, they identified the plasma miRNA candidates in pretreatment samples of DLBCL patients, showing miR-326 and miR-375 as the best markers differentiating B-cell lymphomas from controls. Subsequently, they validated the identified miRNA signature in a confirmatory cohort comprising samples from blood donors, who later developed different types of B-cell lymphoma to test pre-diagnostic plasma samples for early diagnosis. In the end, they verified that both miR-326 and miR199a-5p were upregulated months to years before diagnosis, which showed that changes in plasma miRNAs may precede the development of symptoms of NHL [126].

Circulating miRNAs have also been studied as refiners of the World Health Organization (WHO) classification, leading the way towards a molecular classification of NHL. In fact, the current system of differential diagnosis between the different types of B-cell NHL remains ineffective. For example, Chen et al. demonstrated that plasma levels of miR-21 were higher in DLBCL activated B-cell-like (ABC) compared to the germinal center B-cell-like (GCB), while Bedewy et al. reported higher expression of miR-155 in non-GCB compared to the GCB subtype [112,118]. Moreover, although involving the analysis of FFPE tumor tissues, a study by Leich et al. showed that miRNA expression was different between t(14;18)–negative FLs (without high expression of BCL2) and t(14;18)–positive FL patients. The most robust expression change was verified in miR-16-5p, which was significantly downregulated in t(14;18)–negative FL patients [143]. Similarly, BCL2^+^/BCL6^+^ FL and BCL2^−^/BCL6^+^ FL patients showed upregulation of 21 miRNAs and downregulation of 12 miRNAs compared to BCL6^−^ patients [144]. A major study by Lisio et al. performed a comparative analysis that included all major B-cell NHL types and identified a 128-miRNA signature capable of characterized lymphoma neoplasms, reflecting the lymphoma type, cell of origin, and/or discrete oncogene alterations [145]. Even though these studies do not involve the analysis of circulating miRNAs, they underline the differential molecular background of these NHL subtypes and pave the way to future analysis and validation of these results in the context of liquid biopsies in a larger patient cohort.

Concerning the deregulated expression of lncRNAs in NHL, the vast majority of studies were performed on tissue samples; however, the found tissue-derived deregulated lncRNAs could represent promising circulating biomarkers to be analyzed in future studies. Verma et al. performed a large RNA-seq study of 116 DLBCL tissue samples and identified 2,632 novel lncRNAs, two-thirds of which were only expressed in tumor cells. Moreover, more than one-third of these lncRNAs were found to be differently expressed between ABC and GCB subtypes [146]. LncRNAs PEG10 and LUNAR1 were found to be upregulated in DBCL patients compared to healthy individuals, with areas under the ROC curve (AUC) up to 0.8228 and 0.9420, respectively, indicating their potential diagnostic value [86,128]. Other reported upregulated lncRNAs in DLBCL tissue and cell lines were the lncRNAs FIRRE, HULC, LINC01857, and OR3A4 [76,77,94,129]. A 5-lncRNA signature (upregulated ENST00000424690, ENST00000425358, and NR_026892, and downregulated ENST00000464929 and ENST00000475089) was validated by Gao et al., which distinguished GCB-DLBCL tissue samples from reactive lymph nodes [130]. Moreover, Zhou et al. defined a 17-lncRNA signature, termed SubSigLnc-17, comprising ENTPD1-AS1, SACS- AS1, SH3BP5-AS1, RP11-101C11.1, AC009892.10, RP1- 68D18.4, MIR600HG, RP11-278 J6.4, RP11-203B7.2, CSMD2-AS1, CTC-467 M3.1, RP4-788P17.1, RP11-553 L6.5, CRNDE, RP11-519G16.3, RP11-21 L19.1, and MME- AS1, which permitted the discrimination between GCB and ABC subtypes with high accuracy [131]. In this manner, NONHSAG026900 showed higher levels in the GCB subtype compared to the non-GCB subtype, also indicating the diagnostic potential of these lncRNA [132]. In Deng et al.’s study, the NEAT1_1 expression level was found to increase in DLBCL compared to lymphadenitis [133]. Dousti et al. performed an in silico analysis and identified 189 deregulated lncRNAs in DLBCL, highlighting GAS5, MIR17HG, HULC, and PCA3 with the greatest deregulation score [134]. Pan et al. performed a microarray analysis to identify the expression profile of FL compared to reactive lymphatic nodes. They found 10 altered lncRNAs, with RP11-625 L16.3 being the most significantly upregulated [137]. On the other hand, Roisman et al. performed an RNA-seq analysis in different histological variants of FL and observed that FL3B-DLBCL/tDLBCL showed a higher number of differentially expressed lncRNAs and reported lncRNA RP4-694A7.2 as the most deregulated [138].

Regarding circulating NHL-associated lncRNAs, there are still few studies, even though their diagnostic and prognostic potential hs been shown in several solid tumors [147,148]. The first study on circulating lncRNAs in B-cell lymphoma was performed by Isin et al., which showed downregulation of lincRNA-p21 in CLL patients, upregulation of TUG1 and downregulation of MALAT1, HOTAIR, and GAS5 in MM patients [141]. On the other hand, Wang et al. investigated the circulating lncRNA levels in DLBCL and reported lower levels of lncRNA PANDA in patients’ plasma samples, whereas TUG1 was found upregulated compared to healthy controls [75]. In MCL, LINK-A lncRNA plasma levels were found to significantly increase while lncRNA GATA6-AS was downregulated, allowing to sensitively distinguished patients from healthy controls (AUC of 0.8338 and 0.9195, respectively) [139,149]. Recently, Senousy et al. reported that plasma levels of HOTAIR and XIST were significantly upregulated, whereas GAS5 were downregulated when comparing DLBCL patients to healthy individuals [135]. Interestingly, Tang et al. conjugated the analysis of two circulating ncRNAs, miR-16 and lncRNA MORT, and showed that not only the expression levels of miR-16 and MORT were significantly lower in patients with early-stage MCL compared to controls, but they also showed a regulatory association between the two ncRNA involved in cell proliferation and apoptosis [140].

### 3.2. MiRNAs and lncRNAs as Prognostic Biomarkers

Several studies have been exploring the value of circulating miRNAs as prognostic markers for NHL. In 2008, Lawrie et al. were the first to report that high serum levels of miR-21 were associated with relapse-free survival (RFS) in DLBCL patients, which were later on supported in other studies [110,118]. Similarly, high serum levels of miR-22 at diagnosis in DLBCL were associated with a worse progression-free survival (PFS), independently of the currently used clinical prognostic index [121]. The upregulation of circulating miR-155 and miR-125b was associated with shorter overall survival (OS) of DLBCL patients, while miR-20a/b, miR-93, and miR-106a/b plasma profiles were associated with higher mortality in DLBCL [113,117,124]. Song et al.’s study reported that elevated levels of miR-224, miR-520d-3p, and miR-1236 and lower levels of miR-33a and miR-455-3p were associated with lower medium remission time, and consequently, higher probability of remission, independently of IPI score [122]. A 4-miRNA expression profile (higher levels of miR-21, miR-130b, miR-155; lower levels of miR-28) was shown to be associated with relapse, as well as inferior PFS and OS after R-CHOP, independently of IPI score [114].

The 6-lncRNA signature defined by Sun et al. was shown to be associated with patients OS, independently of standard clinical factors, and permitted to stratify DLBCL patients in high and low-risk groups, improving survival prediction [136]. Moreover, the SubSigLnc-17 profile was not only able to discriminate clinically molecular DLBCL subtypes but also was shown to be significantly associated with patients’ OS and PFS [131]. The expression levels of lncRNA HOTAIR were not only associated with tumor size and clinical stage but also with the presence of B symptoms and IPI score. In fact, higher levels of HOTAIR were associated with better patients’ prognoses, being characterized as an independent predictive biomarker for DLBCL [80,135]. Peng et al. showed in different studies that lncRNAs HULC, LUNAR1, and PEG10 could represent potential prognostic biomarkers of DLBCL. Specifically, HULC and LUNAR1 expression levels were associated with poor prognosis and represented an independent factor of OS and PFS, while PEG10 showed potential as an independent predictor of poor OS of DLBCL [76,86,128]. Moreover, the upregulation of lncRNAs FIRRE and OR3A4 in DLBCL tissue samples is associated with decreased OS [77,129]. In MCL, the tissue-derived MALAT1 and plasma-derived FOXP4-AS1 levels were found correlated with higher MIPI scores and unfavorable patients’ OS and disease-free survival (DFS) [83,93]. Conversely, lower PANDA expression levels, either in plasma or in tissue samples, were associated with inferior RFS and OS of DLBCL patients, being an independent prognostic factor [75]. Moreover, patients expressing high levels of LincRNA-p21 and NONHSAG026900 were found to have a favorable prognosis, with longer OS and PFS, and NONHSAG026900 act as an independent factor by also enhancing the predictive ability of the IPI score [132,150].

The assessment of patients’ prognosis and decisions on treatment alteration are mostly based on imaging PET-CT and clinical evaluations [151]. However, due to the insufficient sensitivity and specificity of these tools, there is an impending need to identify new predictors that permit the early identification of patients with inherent or acquired the refractory disease during treatment. Detection of miRNAs or lncRNAs can lower the detection limit of disease beyond the capabilities of current methods and create a “window of opportunity” for intervention prior to clinical relapse. Earlier initiation of second-line therapy at a point of minimal tumor burden may improve patients’ outcomes. Song et al. identified a 5-miRNA profile (miR-224, miR-455-3p, miR-1236, miR-33a, and miR-520d-3p) associated with R-CHOP response in DLBCL patients, being a significant predictor of response, independent from the IPI score [122]. Specifically, high levels of miR-455-3p and miR-33a were associated with chemosensitivity, while high levels of miR-224, miR-1236, and miR-520d-3p were associated with chemoresistance [122]. Dynamic monitorization of the levels of two potential drug-resistant miRNAs, miR-125b and miR-130a, showed that they are involved not only in recurrence and disease progression but also in chemoresistance in DLBCL patients [124]. Another study took a different approach by analyzing the circulating kinetics of two miRNAs, miR-494 and miR-21, in comparison to interim-PET/CT scans. In this study, they reported that both miRNAs were upregulated in untreated patients and that their levels decreased in patients that achieved interim-PET/CT negativity after four cycles of R-CHOP compared to those that presented a positive interim-PET/CT [119]. Bouvy et al. analyzed the circulating miRNA profile during the treatment course and found higher levels of miR-21 and miR-197 in patients unresponsive to treatment, and high levels of miR-19b, miR-20a, and miR-451 in patients with complete remission, which allowed the differentiation of patients with residual disease from those with complete remission during follow-up [152]. Recently, Fajardo-Ramirez et al. reported a miRNA signature composed of nine upregulated and six downregulated associated with chemoresistance to the R–CHOP regime [153]. Considering these results, circulating miRNAs have a strong potential to be used as predictive and monitoring biomarkers of therapy response in NHL patients. However, further investigation is needed in order to obtain more reliable data.

Regarding lncRNAs as treatment response biomarkers, Senousy et al. observed that pretreatment circulating levels of HOTAIR were higher, whereas GAS5 were lower in non-responders compared to responders to R-CHOP. Moreover, when performing multivariate analysis, HOTAIR appeared as an independent predictor of R-CHOP failure [135]. DLBCL patients with higher expression levels of NONHSAG026900 were shown to have a better response to chemotherapy compared to patients with lower levels [132]. Currently, it is established that treatment failure and consequent relapse can be due to the existence of drug-resistance cancer cell subpopulations [2]. In this instance, chemotherapy-resistant lymphoma cell lines show higher levels of lncRNA MALAT1, which seems to be associated with the inhibition of the autophagy signaling pathway [84]. Using RNA sequencing, Karstensen et al. analyzed the transcription profile of rituximab-sensitive and resistant DLBCL cell lines. They observed a differential expression of lncRNA between sensitive and resistant cells, comprising 54 up and 69 downregulated lncRNAs [154]. In MCL cells, a study by Mourtada-Maarabouni et al. demonstrated that lncRNA GAS5 regulates the effect of known mTOR inhibitors, such as rapamycin, everolimus, and temsirolimus. Particularly, the inhibition of GAS5 substantially decreased the effect of each rapalogue on MCL cells [155]. In Burkitt lymphoma cells, the upregulation of lncRNA MCM3AP-AS1 induced chemotherapy resistance to doxorubicin treatment by regulating the expression of antiapoptotic factors via the miR-15a/EIF4E axis [156]. There are still few studies analyzing lncRNAs as potential biomarkers for treatment response in B-cell lymphomas compared to some solid tumors, with only one studying a circulating lncRNA profile differentiating responders from non-responders.

### 3.3. Clinical Trials for Potential miRNA and lncRNA Biomarkers

Since 2008, when Lawrie et al. demonstrated for the first time the potential of miRNAs as biomarkers in DLBCL patients, a considerable number of clinical trials have been registered to clinically validate them [110]. A clinical trial (NCT01505699) involving 186 patients with B-cell acute lymphoblastic leukemia analyzed microRNA signature levels and their association with different clinical outcomes [157]. On the other hand, clinical trial NCT01057199 specifically tested miR-34a and miR-194 as biomarkers in cell samples from patients with acute myeloid leukemia [158]. Moreover, clinical trial NCT01541800 especially focused on pediatric cancers (central nervous system tumors, leukemia, and lymphoma), evaluated the presence of circulating miRNAs in the blood and cerebrospinal fluid of patients under chemotherapy [159]. Similarly, clinical trial NCT02791217 was developed to identify circulating miRNAs associated with early diagnosis of aggressive hematological malignancies (including B-cell lymphoma) in order to ultimately improve patients’ prognosis and increase survival rates [160]. Another example is the clinical trial NCT03340155, where miRNA expression levels, including miR-155, were tested in the serum of patients diagnosed with skin diseases, such as cutaneous T cell lymphoma (CTCL), and treated with photo(chemo)therapy [161]. One of their objectives was to correlate miRNA levels with clinical responses to treatment at different time points and ultimately identify potential predictive biomarkers. Lastly, clinical trial NCT01606605 included a retrospective and observational analysis of 350 patients diagnosed with DLBCL to study miRNA expression patterns and their correlation with clinical outcome, relapse, and disease progression [162].

Currently, there is no clinical trial exploring the possible application of lncRNAs as lymphoma biomarkers; however, there are some lncRNAs undergoing clinical trials or being patented for solid tumors, such as lung, colorectal, thyroid, breast, and gynecologic cancers [163,164]. To date, the lncRNA PCA3 was the first and only lncRNA approved for clinical practice as an early diagnostic biomarker for prostate cancer [165]. There is only one registered clinical trial, currently active, for hematological cancer to study the correlation between lncRNA XIST and immunophenotyping AML patients (NCT04288739) [166]. Moreover, few clinical trials have been exploring the possible application of combined ncRNA profiles, especially miRNAs and lncRNAs, as biomarkers for the diagnosis and prognosis of cancer [164].

## 4. Conclusions

B-cell NHLs are characterized as a highly heterogeneous group, which is reflected by variability in prognosis and treatment responses between the different subgroups. Therefore, it is crucial to focus on elucidating the molecular background and pathogenesis underlying these malignancies. As previously discussed, in the last years, miRNAs and lncRNAs have emerged as powerful regulators of lymphomagenesis; however, our current knowledge is still limited since it derives essentially from individual research studies of miRNA and lncRNA expression profiles. Additional research is needed to unravel the complex functional network surrounding the miRNAs and lncRNAs and their potential regulatory interactions involved in the process of B-cell lymphomagenesis.

The better understanding we have of the involved molecular mechanisms, the faster we will open clinical avenues toward personalized medicine and the development of new effective therapies, with great clinical benefit for the patient. Moreover, the introduction of miRNAs and lncRNAs as a liquid biopsy strategy provides a great opportunity for improving the diagnostic accuracy and for dynamically monitoring NHL patients in a non-invasive and reproducible manner. Ideally, the expression analysis of a given miRNA/lncRNA, miRNA or lncRNA panel, or even a combination of miRNAs and lncRNAs, should be able to molecularly diagnose and classify patients or predict survival, relapse, remission, and even responsiveness to treatment upfront.

To date, the majority of the studies focus on miRNAs, and there is still a lack of studies on lncRNAs in lymphomas, especially circulating lncRNAs, which opens an interesting opportunity to invest in further understanding their biology and function and their association with miRNAs, and the contribution of lncRNAs profiling in the diagnosis and prognostication of lymphomas.

## Figures and Tables

**Table 1:** Circulating miRNAs as Potential Diagnostic and Prognostic Biomarkers of B-NHL.

NHL	miRNA	Expression	Biomarker Utility	Source Material	Refs.
DLBCL	miR-155	Upregulated	Diagnostic	Serum	[110,111]
			Subclassification	Serum	[112]
			Prognostic of OS, PFS and RFS	Plasma, serum	[113,114]
	miR-210	Upregulated	Diagnostic	Serum	[110,115]
	let-7b/c	Upregulated	Diagnostic	Serum	[116]
	miR-15a	Upregulated	Diagnostic	Serum	[111,115,116]
	miR-16-1	Upregulated	Diagnostic	Serum	[111]
	miR-18a	Upregulated	Diagnostic	Serum	[116]
	miR-20a/b	Upregulated	Prognostic of OS	Serum	[117]
	miR-21	Upregulated	Diagnostic	Serum	[110,115]
			Subclassification	Serum	[118]
			Monitoring	Plasma	[119]
			Prognostic of OS, PFS and RFS	Serum	[110,114,118,120]
	miR-22	Upregulated	Prognostic of PFS	Serum	[121]
	miR-24	Upregulated	Diagnostic	Serum	[116]
	miR-28	Downregulated	Prognostic of OS, PFS and RFS	Serum	[114]
	miR-29c	Upregulated	Diagnostic	Serum	[111]
	miR-33a	Downregulated	Prognostic of RFS	Serum	[122]
	miR-34	Downregulated	Diagnostic	Serum	[111]
	miR-92a	Downregulated	Diagnostic	Plasma	[123]
			Monitoring		
	miR-93	Upregulated	Prognostic of OS	Serum	[117]
	miR-106a/b	Upregulated	Prognostic of OS	Serum	[117]
	miR-125b	Upregulation	Prognostic of OS	Serum	[124]
	miR-130a	Upregulated	Monitoring	Serum	[124]
	miR-130b	upregulation	Prognostic of OS, PFS and RFS	Serum	[114]
	miR-181-5p	Downregulated	Subclassification	Serum	[112]
	miR-199-5p	Upregulated	Prognostic of OS	Plasma	[113]
	miR-224	Upregulated	Prognostic of RFS	Serum	[122]
	miR-323b	Downregulated	Diagnostic	Serum	[125]
	miR-326	Upregulated	Diagnostic	Serum	[126]
	miR-375	Downregulated	Diagnostic	Serum	[126]
	miR-431	Downregulated	Diagnostic	Serum	[125]
	miR-455-3p	downregulated	Prognostic of RFS	Serum	[122]
	miR-494	upregulated	Monitoring	Plasma	[119]
	miR-520d-3p	Upregulated	Prognostic of RFS	Serum	[122]
	miR-1236	Upregulated	Prognostic of RFS	Serum	[122]
CLL	miR-34a	Upregulated	Diagnostic	Serum	[127]
	miR-31-5p	Upregulated	Diagnostic	Serum	[127]
	miR-150-5p	Upregulated	Diagnostic	Serum	[127]
	miR-155-5p	Upregulated	Diagnostic	Serum	[127]
	miR-15a-3p	Upregulated	Diagnostic	Serum	[127]
	miR-29a-3p	Upregulated	Diagnostic	Serum	[127]

Abbreviations: CLL—Chronic lymphocytic leukemia; DLBCL—Diffuse large B-cell lymphoma; OS—Overall Survival; PFS—Progression-free Survival; RFS—Relapse-free Survival.

**Table 2:** LncRNAs as Potential Diagnostic and Prognostic Biomarkers of NHL.

NHL	LncRNA	Expression	Biomarker Utility	Source Material	Refs.
DLBCL	PEG10	Upregulated	Diagnostic	Tissue Cell lines	[128]
			Prognostic of OS		
	LUNAR1	Upregulated	Diagnostic	Tissue Cell lines	[86]
			Prognostic of OS and PFS		
	FIRRE	Upregulated	Diagnostic	Tissue Cell lines	[77]
			Prognostic of OS		
	HULC	Upregulated	Diagnostic	Tissue Cell lines	[76]
			Prognostic of OS and PFS		
	LINC01857	Upregulated	Diagnostic	Tissue Cell lines	[94]
	OR3A4	Upregulated	Diagnostic	Tissue Cell lines	[129]
			Prognostic of OS		
	ENST00000424690	Upregulated	Diagnostic	Tissue Cell lines	[130]
	ENST00000425358				
	NR_026892				
	ENST00000464929	Downregulated			
	ENST00000475089				
	SubSigLnc-17	-	Diagnostic	Tissue Cell lines	[131]
			Subclassification		
			Prognostic of OS and PFS		
	NONHSAG026900	Upregulated	Diagnostic	Tissue Cell lines	[132]
			Prognostic of OS and PFS		
	NEAT1_1	Upregulated	Diagnostic	Tissue Cell lines	[133]
			Prognostic of OS		
	GAS5	Upregulated	Diagnostic	Tissue Cell lines	[134]
	MIR17HG	Upregulated	Diagnostic	Tissue Cell lines	
	HULC	Upregulation	Diagnostic	Tissue Cell lines	
	PCA3	Upregulated	Diagnostic	Tissue Cell lines	
	PANDA	Downregulation	Diagnostic	Plasma Tissue	[75]
			Prognostic of OS and RFS		
	TUG1	Upregulated	Diagnostic	Plasma	[75]
	HOTAIR	Upregulated	Diagnostic	Plasma Tissue	[80,135]
			Predictive of Treatment response		
			Prognostic of OS		
	XIST	Upregulated	Diagnostic	Plasma	[135]
	GAS5	Downregulated	Diagnostic	Plasma	
			Predictive of Treatment response		
	6-lncRNA signature	-	Prognostic of OS	Tissue	[136]
FL	RP11-625 L16.3	Upregulated	Diagnostic	Tissue	[137]
	RP4-694A7.2	Upregulated	Diagnostic and subclassification	Tissue	[138]
MCL	LINK-A	Upregulated	Diagnostic	Plasma	[139]
	GATA6-AS	Downregulated	Diagnostic	Plasma	
	MALAT1	Upregulated	Prognostic of OS and DFS	Tissue Cell lines	[83]
	FOXP4-AS1	Upregulated	Prognostic of OS and DFS	Plasma	[93]
	MORT	Downregulated	Diagnostic	Plasma	[140]
CLL	lincRNA-p21	Downregulated	Diagnostic	Plasma	[141]
MM	TUG1	Upregulated	Diagnostic	Plasma	
	MALAT1	Downregulated	Diagnostic	Plasma	
	HOTAIR		Diagnostic	Plasma	
	GAS5		Diagnostic	Plasma	

Abbreviations: CLL—Chronic lymphocytic leukemia; DFS—Disease-free Survival; DLBCL—Diffuse large B-cell lymphoma; FL—Follicular lymphoma; MCL—Mantle cell lymphoma; MM—Multiple Myeloma; OS—Overall Survival; PFS—Progression-free Survival; RFS—Relapse-free Survival.
